# Information exchange networks of health care providers and evidence-based cardiovascular risk management: an observational study

**DOI:** 10.1186/s13012-016-0532-1

**Published:** 2017-01-13

**Authors:** Naomi Heijmans, Jan van Lieshout, Michel Wensing

**Affiliations:** 1 Department IQ healthcare, Radboud Institute of Health Sciences, Radboud University Medical Centre, PO 9101 6500 HB, Nijmegen, The Netherlands; 2 Department of General Practice and Health Services Research, Heidelberg University Hospital, Marsilius Arkaden-Turm West, Im Neuenheimer Feld 130.3, 69120 Heidelberg, Germany

**Keywords:** Social network analysis, Evidence-based practice, Primary care, Cardiovascular disease, Implementation science

## Abstract

**Background:**

Although a wide range of preventive and clinical interventions has targeted cardiovascular risk management (CVRM), outcomes remain suboptimal. Therefore, the question is what additional determinants of CVRM and outcomes can be identified and addressed to optimize CVRM. In this study, we aimed to identify new perspectives for improving healthcare delivery and explored associations between information exchange networks of health care providers and evidence-based CVRM.

**Methods:**

This observational study was performed parallel to a randomized clinical trial which aimed to improve professional performance of practice nurses in the Netherlands. Information exchange on medical policy for CVRM (“general information networks”) and CVRM for individual patients (“specific information networks”) of 180 health professionals in 31 general practices was measured with personalized questionnaires. Medical record audit was performed concerning 1620 patients in these practices to document quality of care delivery and two risk factors (systolic blood pressure (SBP) and LDL cholesterol level). Hypothesized effects of five network characteristics (density, frequency of contact, centrality of CVRM-coordinators, homophily on positive attitudes for treatment target achievement, and presence of an opinion leader for CVRM) constructed on both general and specific information exchange networks were tested and controlled for practice and patient factors using logistic multilevel analyses.

**Results:**

Odds for adequate performance were enhanced in practices with an opinion leader for CVRM (OR 2.75, *p* < .05). Odds for achievement of SBP targets were reduced in practices who had networks with low homophily on positive attitudes for SBP and LDL targets (homophily for SBP targets OR 0.57, *p* < .05 and OR 0.60, *p* < .05, homophily for LDL targets OR 0.59, *p* < .05 and OR 0.61, *p* < .05 in general and specific information networks, respectively). No effects of network characteristics on cholesterol were found.

**Conclusions:**

Delivery of evidence-based CVRM is associated with homophily of clinical attitudes and presence of opinion leaders in primary care teams. These results signal the potential of social networks to be taken into account in further attempts to improve the implementation of evidence-based care for CVRM. Future research is needed to identify and formulate optimal strategies for using opinion leaders to improve CVRM. Future interventions may be more effective if they target a common vision on CVRM within practices.

## Background

Although examples of successful change of healthcare practice exist, there is a need for additional approaches that are more consistently effective. Determinants of evidence-based practice to which implementation programs can be tailored may be identified by social network analysis. Social networks are important channels for information exchange and coordination of activities, which are both influenced by network structures and cultures [1]. Social network analysis in health care has been used to describe and explore a range of processes in healthcare, such as social support of patients, collaboration of health professionals, and the uptake of new practices [2]. The importance of social networks for health care delivery is illustrated by studies showing, for example, that interaction and communication patterns among health care providers can be crucial to improve patient safety [3], and coordination and quality of care [3, 4]. In this study, we explore the role of information exchange networks of primary care providers in the delivery of evidence-based cardiovascular risk management (CVRM).

### Practical context

Cardiovascular disease (CVD) remains an important cause of mortality and reduced quality of life worldwide [5]. CVD was the number one cause of death among women and the second cause of death for men in the Netherlands in 2013 [6]. A range of preventive and clinical interventions are recommended in patients with CVD or high vascular risk. Clinical practice guidelines emphasize the importance of comprehensive CVRM, life style changes, and preventive drug therapy [7]. In the Netherlands, organizational and financial conditions for providing recommended CVRM have been optimized in recent years. Among these are the publication of a multidisciplinary clinical guideline and organizational standards for general practices, the introduction of nurses in practices, nationwide supply of paper-based and online patient education tools for CVD patients as well as the general public, and targeted reimbursement for chronic illness care in primary care [8]. Although the quality of CVRM improved substantially, still a specific number of patients did not completely receive recommended CVRM or did not reach target values of CVRM [9]. There is a need for new approaches to enhance evidence-based CVRM.

### Theoretical background

Literature on social network analysis is expanding and has provided descriptions of social network structure, or the pattern of connections between individuals, and of network culture, e.g., shared values, beliefs, or interests of individuals who are connected. Of these, a number of network characteristics were selected which were expected to be related to the implementation of evidence-based care for CVRM.

*Network density* describes the proportion of all possible connections in a given network that are present and has been used as an indicator of group solidarity or cohesion [1]. In dense networks, many members know each other and interact with each other frequently. The multiplicity of ties creates opportunities for various social influence processes, such as social comparison, imitation of successful behavior [1, 10, 11], and the setting of group norms [1]. High density has been related to fast diffusion of information [12] and has been shown to improve tasks that depend on cooperation [13] and coordination performance [14].

A high *frequency of contact*, expected to be present in dense networks because of their multiplicity of connections, can be of importance for health care delivery, as it enhances opportunities for social influence which, in turn, can offer protection against egocentric choices [11, 15, 16]. The underlying mechanism is derived from a game theory, which distinguishes between single episode and repeated interactions. Experiments based on a game theory showed that the dynamics of repeated contacts provided a context facilitating and enhancing development of long-term cooperation and trust [17, 18]. In this view, outcomes depend on the history of contact between individuals, and cooperative and trustworthy behaviors are being incentivized by the anticipation of long-term reciprocal benefits [18, 19].

Network members with high *centrality* have many connections with others in the network. These individuals are expected to be influential as their number of connections allows for greater access to and control over resources [20]. High centrality has been associated with enhanced knowledge transfer [21, 22]. In CVRM in primary care in the Netherlands, individuals with high centrality are expected to be present in social networks as CVRM coordinators or case managers. Both are purposefully created to become highly central individuals in health care delivery networks.

*Homophily*, or homogeneity, is the tendency of individuals with similar characteristics to associate and bond with each other. This concept refers to the tendency of persons to assume that individuals similar to them are more likely to accept them, to be trustworthy, and have similar beliefs. As such, homophily can be considered to be a social heuristic, which aims to avoid risks of connecting with others, e.g., by prevention of potential conflicts and misunderstandings and by monitoring the balance of benefits and costs of relations [23, 24]. High homophily may enhance uptake of information which spreads in a given network by mutual reinforcement of attitudes and behaviors [24]. Social networks can be homogenous on several attributes. One study showed that physicians were more likely to exchange information and to provide advice during patient treatment if their attitudes towards evidence-based medicine were similar, if they had the same specialty, worked in the same organization, and had co-authored peer-reviewed papers [25]. Homophily has been related to medical advice seeking of clinical staff [26] and prescribing behavior of general practitioners [20].

Social networks may contain informal *opinion leaders*. He or she represents a person who influences opinions, attitudes, beliefs, motivations, and behaviors of others [27]. The role is informal, because it is not necessarily linked to a position in a formalized organization. Opinion leaders may be beneficial for promoting evidence-based practice [28] as their presence has been related to speeding adoption of clinical guidelines [29] and to adherence to guidelines for unstable angina [30].

These network characteristics may be considered to relate to two broad dimensions of social networks. Density, frequency of contact, and centrality provide descriptions of patterns of linkages between actors in networks, which together describe the network structure in which information and other commodities are transferred. Homophily and presence of opinion leaders relate to shared opinions and existing values and norms in networks, in other words, shared views on the world. This distinction is based on general conceptualizations of structure and culture (e.g., in [31, 32]). It should be noted that structure and culture influence each other (for example see [33]).

**Fig. 1:**
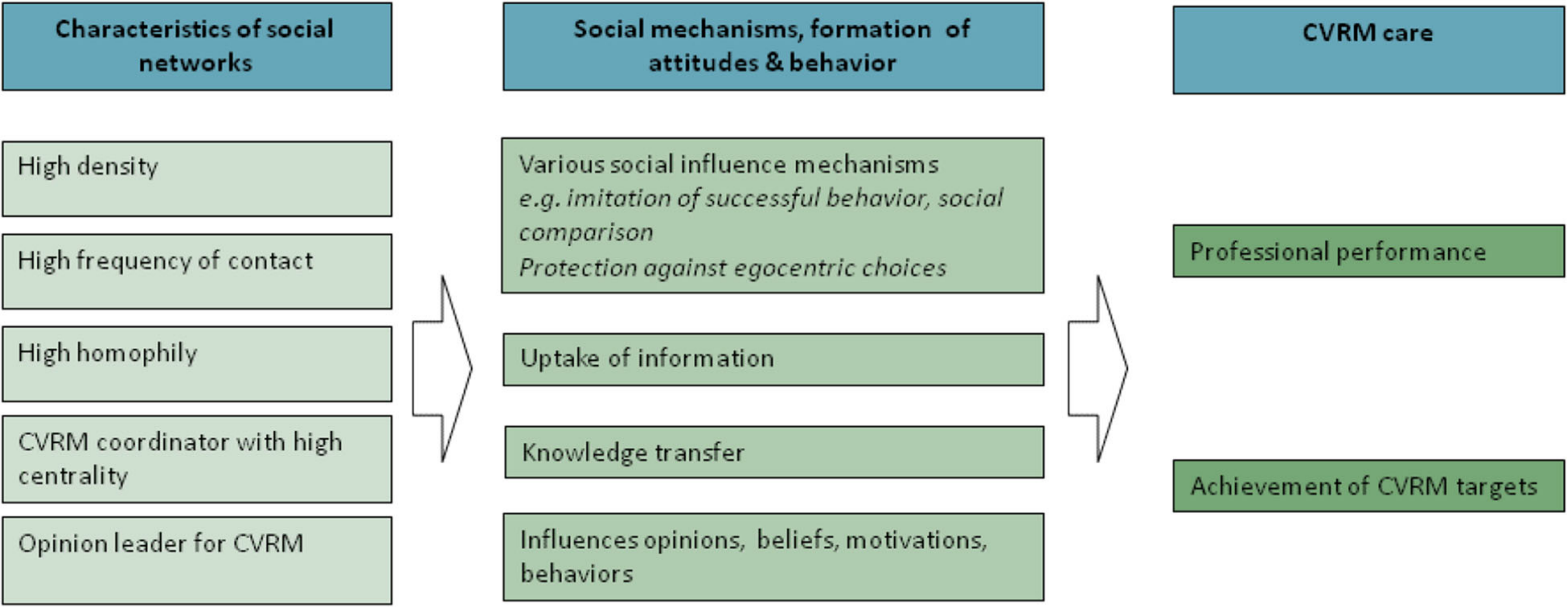
Hypothesized relations

This study focused on network characteristics of healthcare professionals in general practices. The aim of the study was to explore associations of network density, frequency of contact, centrality of coordinators, homophily, and presence of informal opinion leaders with aspects of quality of care delivery and clinical risk factor levels of patients. We expected that patients are more likely to receive evidence-based CVRM and reach CVRM targets in practices which have social networks characterized by high density, high frequency of contact, a CVRM-coordinator who has a high degree of centrality, high homophily on positive attitudes for achievement of treatment targets, and a consistently identified opinion leader for CVRM (Fig. 1).

## Methods

### Design

This study was part of the Tailored Implementation for Chronic Diseases (TICD) project [34] and was an observational study on information exchange networks of health care professionals involved in CVRM. The study was performed parallel to a larger two-arm cluster randomized controlled trial (RCT), which was also part of the TICD project [35]. RCT’s main aim was to test a tailored intervention for improving CVRM in primary care by enhancing professional performance of the practice nurses [36]. The practice nurses have an important role in CVRM and are responsible for providing patients with consults, including advice on their self-management behaviors. The current study was an independent observational study, not a process evaluation of the trial.

### Ethical approval

The Medical Ethical Committee of Radboud University Nijmegen Medical Centre has waived approval for both the network study [36] and the RCT [35].

### Study population

The sample of the social network study consisted of health care professionals and patients with high risk for CVD and established CVD.

#### Health care professionals

We included all health care professionals working in general practices participating in the RCT and who were involved in patient care. These included general practitioners, general practitioners in training, locum general practitioners, dispensing general practitioners, the practice nurses (specialized in somatic and in mental health care), practice assistants, pharmacist assistants, and social workers.

#### Patients

Eligible patients were adults aged 18 or older, with a high risk of CVD or established CVD and capable of providing informed consent. Patients with high risk for CVD have a risk score of 20% or higher of 10-year-morbidity and mortality due to CVD. International Classification of Primary care (ICPC) codes were used to extract eligible patients from medical records. Exclusion criteria consisted of diabetes mellitus, pregnancy and lactation, terminal illness, cognitive impairments, and poor language skills.

### Data collection procedures

Data on information exchange networks were collected using questionnaires, which had been successfully applied in previous research [37–39]. Questionnaires were personalized and listed names of all persons involved in patient care in the participating practices. Personalizing was performed by deriving names online, which were checked for accuracy by the practice nurses. Questionnaires, along with prepaid envelopes for returning questionnaires, were distributed to the practice nurses during outreach visits, performed for purposes of the RCT at the start of its program. The practice nurses distributed the network questionnaires to other health professionals within practices and were asked to remind other health professionals in case of no response within 3 weeks. A maximum of two reminders was used.

Data on professional performance of the practice nurses and patient risk factors were gathered from patients’ medical records, using the adapted EPA-Cardio abstraction tool [40], at the end of the RCT intervention period at 6-month follow-up. Medical auditing was performed by trained research assistants. All data collection was performed between July 2013 and September 2014.

### Outcomes and measures

The main outcomes of this study consisted of one measure of quality of care delivery and two specific vascular risk factors as proxies for health outcomes. Quality of care delivery was reflected by professional performance of the practice nurses, which was the target of the larger RCT this study was embedded in.

#### Professional performance

Professional performance reflected application of evidence-based recommendations for personalized counseling and education of CVRM patients by the practice nurses. Professional performance was defined dichotomously, reflecting adequate or inadequate performance. Professional performance was considered adequate when at least one of the following conditions was met:

1. There is a record in the patient’s medical file or other healthcare provider-based records that the patient has received advice on at least one lifestyle item as specified in prevailing guidelines of CVRM (diet, smoking, or physical exercise) and which has been relevant for the individual patient in the previous 6 months. At least one target, made up maximally 15 months ago, for improving an aspect of lifestyle should be recorded. Also, the practice nurses were required to make a register note when a patient has an adequate lifestyle.
2. There is a notation in the patient’s medical file that the patient has none, mild, or major depressive symptoms and that the patient has been referred to E-health, a physical exercise group, or depression treatment, respectively.

#### Patient risk factors

The patient risk factors consisted of systolic blood pressure (SBP) and low-density lipoprotein cholesterol (LDL). Elevated SBP was defined as SBP > 140 mm Hg. Elevated LDL was defined as LDL > 2.5 mmol/l.

Other measures of the study included:

#### Descriptive variables

Descriptive measures included type of practice (solo, duo, or group) and practice size (number of staff); these data were measured using the modified EPA-Cardio abstraction tool [40].

#### Information items for constructing social networks

Information exchange networks were measured using personalized questionnaires for each practice with a roster format. Social networks were constructed in two ways per practice. Health professionals were asked to indicate all their social contacts from their general practice from the last 12 months for CVRM information receiving and providing on two subjects. The first subject was general CVRM, information sharing on medical policy for CVRM in general. The second subject was specific CVRM, information sharing on CVRM related to specific patients. We chose to measure both general and specific information exchange networks as contacts within these networks can be expected to differ. For example, information exchange on CVRM in general relates to most or every health professional within a practice while information exchange related to specific patients may involve mainly health professionals who are involved in the treatment of these individuals.

#### Frequency of contact

Health professionals were asked to indicate whether they had been in contact on a (1) daily/weekly or (2) monthly/yearly basis, for each person they had shared information with.

#### CVRM coordinators

Health professionals were asked to list the name(s) of the person(s) responsible for coordination of CVRM within the particular practice and to name his/her profession.

#### Attitudes on CVRM targets

Health professionals were asked to indicate on a 5-point Likert scale (1 “totally unimportant”–5 “highly important”) how important they considered the treatment targets “achievement of SBP < 140” and “achievement of LDL < 2.5” in patients for whom decisions on appropriate treatment can be considered as debatable. Therefore, it was stated that attitudes involved treatment of patients for whom the guideline for CVRM was applicable, who were elderly (80 years and older) and had a limited life expectancy (less than 5 years). The scores 1–3 were considered as disagreement, and scores 4–5 were considered as agreement with the importance of achievement of SBP and LDL targets.

#### Opinion leaders

Health professionals were asked to provide the name and occupation of one person they considered to have a significant influence on their current practice in CVRM. Additional instructions stated that “this person can be anyone from inside or outside the practice, and that the influence this person has had can be either current or from the past”.

### Data analysis

The statistical package R (package Statnet) was used for constructing and obtaining social network parameters of practices on general and specific information exchange. SPSS (version 22) was used for all other analyses. The primary unit of analysis was practice (one network per practice) except where indicated otherwise.

*Reliability* of reported social network connections was investigated by examining the proportion of all possible connections that were mutually reported present or absent (reciprocity coefficients in non-directed networks). In accordance with guidelines on handling missing values, we substituted *missing values* on information receipt for networks with at least 60% reliability with values as provided by responses of other persons on providing information. In case of no information on connections, we indicated no contact by filling in a zero in the data [41]. Only missing data on connections were imputed. Missing data on attitudes on CVRM targets were not imputed; therefore, persons who did not provide data on attitude variables were left out on the calculation of the E-I index.

#### Construction of network characteristics

Network characteristics hypothesized to be of positive influence were as follows: *a high density*, *high frequency of contact*, *presence of a CVRM*-*coordinator who has a high degree of centrality*, *high homophily*, *and a consistently identified opinion leader for CVRM*. All characteristics except “opinion leader for CVRM” were computed and tested separately for information receipt networks of general practices on (1) CVRM in general and (2) CVRM for specific patients. Presence of opinion leaders was inferred using data from all health professionals from the specific practice, regardless of information exchange.

*Density* represented the proportion of all possible connections in the information exchange network of professionals in a practice organization that were present and was entered as a continuous score in analyses. *High frequency of contact* was indicated by the number of contacts within a practice network occurring on a daily or weekly basis and was entered as a continuous score in analyses. For *presence of a CVRM-coordinator who has a high degree of centrality*, we first determined whether CVRM coordinators were present within practices. In almost all practices, coordinators were present, with up to three persons identified as such. We then determined which person was mentioned most often as coordinator by his or her colleagues and computed his or her centrality (total degree, which is the total number of connections providing the coordinator with information and to which the coordinator provides information) which was entered as a continuous score in analyses. In three practices, two persons, a practice nurse and a general practitioner, received equal votes as coordinators. To term one of them as coordinator of the practice, we considered which type of health professional was mentioned most often as coordinator in other practices. As most practices had a practice nurse as coordinator, it was decided for these three practices to enter centrality scores of the practice nurses in the analyses.

We assessed *homophily* on positive attitudes regarding achievement of treatment goals for SBP and LDL. Homophily was calculated using the E-I index [42]. The E-I index ranges from −1 to 1. When the E-I index is −1, all ties in the networks are between contacts who agree on the importance of achievement of treatment goals (i.e., the network is homophilous in positive attitudes), while a score of 1 indicates that all ties are between contacts who disagree with this importance (the network is homophilous in negative attitudes). A score of 0 indicates that ties in the network are between both contacts with positive and negative attitudes (the network is heterophilous in attitudes).

For testing *a consistently identified opinion leader for CVRM*, we first computed the percentages of votes for each person as opinion leader within the practice. A dichotomous item was then created; practices in which one person was chosen as opinion leader by at least 60% of his colleagues were designated as having an opinion leader. We chose to conceptualize that only one opinion leader could exist in each practice because of their interpersonal influences on opinions. While one opinion leader may exert specific influences, two opinion leaders may spread contrary ideas which may lead to the possible spread of opposing ideas within networks.

#### Statistical analyses

All analyses were performed two tailed, using *p* < .05 indicating significance, and were based on “intention to treat” with practice’s networks as unit of analysis. For comparison of social network characteristics as constructed on networks for general and specific CVRM, paired sample *t* tests (T) were performed for normally distributed characteristics; for non-normal distributed characteristics, Wilcoxon tests (Z) were performed. Network effects from the hypotheses were tested using multivariate logistic regression models, with random intercepts specified for general practice. Professional performance of the practice nurses, and SBP and LDL of patients, measured at 6-month follow-up of the RCT were used as dependent variables.

For each outcome, 11 multivariate models were specified, testing each network effect controlled for patient and practice characteristics. Patient characteristics were entered as level 1 predictors and practice characteristics were entered as level 2 predictors in the analyses. Ten models tested the 5 network effects (density, frequency of contact, centrality of the CVRM coordinator, and homophily for positive attitudes on achievement of treatment targets for SBP and LDL) as constructed on (1) specific and (2) on general information receipt networks. One model for each outcome was used to test the effect of the presence of an opinion leader. Control variables consisted of patients’ characteristics (age, sex, patient group; established CVD versus high risk) and practice characteristics (network size and RCT arm; control or intervention).

## Results

### Response rates and reliability measures

The RCT started with 44 practices, of which 10 dropped out, so that 34 practices completed the intervention program. A total of 37 practices (84%) provided network data. Of these 37, six practices had to be excluded from the analyses. Three practices provided data on their networks but not on the study outcomes as they dropped out of the RCT. Of the remaining 34 practices, three practices completed the RCT but had low response on the network questionnaires (in two practices, only two professionals participated and one practice had less than 40% response). Thus, data on a total of 31 practices were available for the analyses.

From a total of 242 health professionals (from 31 practices), 186 completed network questionnaires so that the response rate was 76.9%. Average data completeness per practice was 79.5% (range 40%–100%). *Reliability* calculated for network connections was 81% (SD 19.7%) for general CVRM and 77.6% (SD 25.8%) for specific CVRM.

### Sample characteristics

From the 31 practices, 14 were randomized to the RCT control arm and 17 to its intervention arm. 18 practices were solo practices, 10 duo, and 3 group practices. 17 practices were situated in a rural area whereas 14 were from an urban area. Mean number of health professionals working within practices was 7.8 (SD 2.9).

The practice nurses (*n* = 31) had a mean age of 42.7 years (SD 8.6), with an average of 11.9 years (SD 10.3) of working experience. Of 1620 patients who participated, 870 (54%) were at high risk for CVD and 750 (46%) had established CVD. The mean age of high-risk patients was 73 years (SD 7.3) and 31% was female. The mean age of CVD patients was 68.7 years (SD 10.9) years and 38% was female.

### Description of social networks

#### Density

The mean density of network connections for general CVRM information in the practices was 0.38 (SD 0.17) and 0.37 (SD 0.22) for specific CVRM information.

#### Frequency of contact

The mean number of total network connections for general CVRM information was 18.16 (SD 11.69), of which 51% (SD 27.37%) were high frequency contacts (contact on a daily or weekly basis). For specific CVRM information, the mean number of total network connections was 17.81 (SD 13.92), of which 61% (SD 23.98%) were high frequency contacts.

#### CVRM coordinator(s)

CVRM coordinators were present in the majority (*n* = 28, 90%) of the general practices; in 4 practices 80%–87.5% of the health professionals reported, a CVRM coordinator was present, and in 24 practices, everyone within the practices agreed on having a coordinator. Three practices had low agreement on having a coordinator, with 25%–50% of health professionals reporting a coordinator was present within their practice.

A single coordinator was present in 8 (29%) practices, 16 (57%) practices reported they had two CVRM coordinators, and 4 practices (14%) had three CVRM coordinators. Consistency of recognition of coordinators seemed to decrease when more persons were identified as coordinator; a single person was recognized as coordinator by all of his or her colleagues in 75% of practices with a single coordinator and in 63% of practices with two coordinators, while recognition by all colleagues was not obtained in practices with three coordinators.

Considering persons most consistently chosen as CVRM coordinator of the practice, most practices’ (*n* = 19, 68%) coordinator was a practice nurse, 6 practices (21%) reported their coordinator was a general practitioner, while in 3 practices (11%), the practice nurses and general practitioners were mentioned equally often as coordinators.

#### Centrality of CVRM coordinators

Centrality scores were computed for persons most often elected as CVRM coordinator. Mean centrality for general CVRM information was 6.61 connections (SD 3.38) and 7.07 connections (SD 4.97) for specific CVRM information.

#### Homophily on attitudes on treatment targets

For achievement of SBP treatment targets, an average number of 1.48 (SD 1.48) health professionals per general practice indicated to consider achievement of such targets important while on average, 3.94 (SD 1.61) health professionals per practice considered achievement of SBP targets as unimportant.

An average number of 1.39 (SD 1.36) health professionals per practice valued achievement of LDL treatment targets while an average of 3.94 (SD 1.61) health professionals disagreed with the importance of achievement of LDL targets.

The mean value of the E-I index in general CVRM information exchange networks was 0.60 (SD 0.43) regarding attitudes for achievement of SBP targets and 0.53 (SD 0.49) on achievement of LDL targets.

The mean value of the E-I index in specific information exchange networks was 0.55 (SD 0.46) for achievement of SBP targets and 0.52 (SD 0.52) for achievement of LDL targets. These values indicate that on average most reported ties were between contacts who did not value the importance of achievement of treatment targets.

### Opinion leaders

In most cases, opinion leaders were persons from within practices, with a mean of 3.7 (SD 1.8) health professionals per practice naming a within practice colleague as opinion leader and a mean of 0.7 (SD 0.97) health professionals per practice mentioning a person from outside the practice as their opinion leader.

In 10 (32%) practices, an opinion leader was consistently identified, as designated by at least 60% of health professionals from practices naming a specific person as their opinion leader. In these practices, most practices (*n* = 8) choose a general practitioner as opinion leader, one practice choose a practice nurse as opinion leader; and in one practice, equal votes were given to a general practitioner and practice nurse as opinion leader.

Considering data of all practices and appointing individuals most often mentioned by his or her colleagues as opinion leader of the practice, a similar pattern was found. In most practices (*n* = 24), a general practitioner was chosen as opinion leader, 3 practices choose a practice nurse as opinion leader, and 3 practices gave equal votes to a general practitioner and practice nurse as opinion leader. In one practice, no opinion leaders from within the practice were found.

#### Comparison of network characteristics

The several network characteristics showed substantial variation between practices (see Table 1). They were not different for networks constructed on general or specific CVRM information exchange (density T 0.33, *p* = .745; number of high frequency contacts Z 1.76, *p* = .079; CVRM coordinator centrality Z 0.643, *p* = .520; homophily on SBP targets Z −1.10, *p* = .272; homophily on LDL targets Z −0.27, *p* = .790) (Table 1).

**Table 1:** Descriptive data of networks

	General CVRM networks				Specific CVRM networks			
	Mean	SD	Min	Max	Mean	SD	Min	Max
Density	0.38	0.17	0.08	0.83	0.37	0.22	0	0.83
Number of high frequency contacts	8.87	5.64	0	20	10.65	8.24	0	33
Centrality of CVRM coordinator	6.61	3.38	2.00	16	7.07	4.97	0	24
Homophily on SBP targets	0.60	0.43	−0.42	1.00	0.55	0.46	−0.46	1.00
Homophily on LDL targets	0.53	0.49	−0.87	1.00	0.52	0.52	−0.87	1.00

### Hypotheses testing

#### Network characteristics and professional performance

General practices with consistently identified opinion leaders had increased odds for adequate professional performance of the practice nurses (OR 2.75, *p* < .05). None of the other network characteristics constructed on either general or specific information networks were related to professional performance.

Results for control variables in models for both general and specific CVRM were as follows. In each model, reduced odds for adequate professional performance were found for patient age and CVD patients, while female patients had enhanced odds for adequate performance. No effects of network size or trial arm were found (Table 2).

**Table 2:** Network characteristics and professional performance

Professional performance	General CVRM			Specific CVRM				
	OR	95% CI		OR	95% CI		*n* prac	*n* pat
Density	9.51	0.62	145.6	4.30	0.57	32.36	31	1620
Frequency of contact	1.03	0.96	1.11	1.03	0.96	1.11	31	1620
Centrality of CVRM coordinator	1.03	0.90	1.18	1.01	0.11	1.01	28	1462
Homophily								
Achieve BP target	0.69	0.26	1.78	0.72	0.29	1.83	30	1583
Achieve LDL target	0.71	0.30	1.65	0.73	0.32	1.65	30	1583
Consistently identified OL for CVRM	2.75*	1.23	6.14				31	1620

Not shown in table estimates for control variables, estimates for intercepts, and estimates for random effects

*OR* odds ratio, *n prac* number of practices in analysis, *n pat* number of patients in analysis, *OL* opinion leader

*p < .*05*

### Network characteristics and blood pressure

#### General CVRM information networks

Negative associations were found between homophily and recorded blood pressure. Homophily was measured using the E-I index, ranging from −1 (positive attitudinal homophily; all contacts are between health professionals who consider achievement of treatment targets important), 0 (contacts are between professionals who value this as well as professionals who do not value this), to 1 (negative attitudinal homophily; all contacts are between health professionals who do not consider achievement of treatment targets as important). The negative coefficient then indicates that when the E-I index increases (i.e., moves towards *negative* attitudinal homophily), the odds for positive SBP outcomes of patients decrease. Stated otherwise, in networks in which homophily on *positive* attitudes for achievement of both SBP targets (OR 0.57, *p* < .05) and LDL targets (OR 0.59, *p* < .05) was low (i.e., networks in which there were few contacts between persons considering achievement of treatment targets as important), reduced odds for positive SBP outcomes were found. None of the other network characteristics constructed on general information receipt networks were related to SBP.

#### Specific CVRM information networks

Low homophily on both positive attitudes for achievement of treatment targets of SBP and LDL were related to reduced odds for positive SBP outcomes of patients (OR 0.60 and OR 0.61, respectively, *p* < .05 for both effects). None of the other network characteristics constructed on specific information receipt networks were related to SBP.

Results for control variables in models for both general and specific CVRM were as follows. In each model, patient age was significantly related to reduced odds for positive SBP outcomes, while CVD patients had enhanced odds for positive SBP outcomes. No effects were found for patient sex, network size, and trial arm (Table 3).

**Table 3:** Network characteristics and blood pressure

SBP	General CVRM			Specific CVRM				
	OR	95% CI		OR	95% CI		*n* prac	*n* pat
Density	1.56	0.31	7.82	1.04	0.33	3.35	31	968
Frequency of contact	1.02	0.98	1.07	1.00	0.97	1.03	31	968
Centrality of CVRM coordinator	1.00	0.93	1.09	1.00	0.94	1.06	28	883
Homophily								
Achieve BP target	0.57*	0.34	0.94	0.60*	0.37	0.98	30/29	943/921
Achieve LDL target	0.59*	0.38	0.92	0.61*	0.40	0.95	30/29	943/921
Consistently identified OL for CVRM	0.98	0.59	1.64				31	968

Not shown in table estimates for control variables, estimates for intercepts, and estimates for random effects

*OR* odds ratio, *n prac* number of practices in analysis, *n pat* number of patients in analysis, *OL* opinion leader

*p < .*05*

#### Network characteristics and serum cholesterol

None of the social network characteristics, constructed on either general or specific information networks, were related to LDL.

Results for control variables in models for both general and specific CVRM characteristics were as follows. In the models testing homophily of SBP and LDL targets, patient age had significant positive effects on positive LDL outcomes while no effects of age were found in the other models. Female patients had significant or marginally significant reduced odds for favorable LDL outcomes in all models, CVD patients had significant enhanced odds for positive LDL outcomes in all models, while no effects were found for network size and trial arm (Table 4).

**Table 4:** Network characteristics and serum cholesterol

LDL	General CVRM			Specific CVRM				
	OR	95% CI		OR	95% CI		*n* prac	*n* pat
Density	0.72	0.16	3.30	0.89	0.30	2.64	31	662
Frequency of contact	0.98	0.95	1.02	0.99	0.97	1.02	31	662
Centrality of CVRM coordinator	0.98	0.92	1.06	1.00	0.95	1.06	28	627
Homophily								
Achieve BP target	1.05	0.63	1.78	0.85	0.52	1.40	30/29	642/625
Achieve LDL target	0.97	0.61	1.54	0.89	0.57	1.38	30/29	642/625
Consistently identified OL for CVRM	1.39	0.88	2.20				31	662

Not shown in table estimates for control variables, estimates for intercepts, and estimates for random effects

*OR* odds ratio, *n prac* number of practices in analysis, *n pat* number of patients in analysis, *OL* opinion leader

*p < .*05*

## Discussion

In this observational study, we explored linkages between information exchange network characteristics of health care providers in general practices and the implementation of evidence-based care for CVRM and vascular risk factors as proxies for health outcomes. Several of our hypotheses were confirmed (see Table 5 for a descriptive summary). Low homophily of positive attitudes on achievement of treatment targets was negatively related to achievement of recommended SBP values of patients. Presence of consistently perceived opinion leaders was positively related to adequate professional performance of the practice nurses. Results for network characteristics of general and specific CVRM information networks were largely similar. No effects were found of network density, frequency of contact, and centrality of coordinators on professional performance and clinical risk factors. Overall, we find some indication of impact for culture in primary care teams, but no indication for impact of network structures.

**Table 5:** Summary of results

	Outcomes for which hypothesis was confirmed	
General practices will have positive outcomes if their network are characterized by:	General CVRM networks	Specific CVRM networks
High density	N.s.	N.s.
High frequency of contact	N.s.	N.s.
Centrality of CVRM coordinator	N.s.	N.s.
Homophily on positive attitudes regarding		
Achievement of BP-targets	SBP	SBP
Achievement of LDL-targets	SBP	SBP
Consistently identified OL for CVRM	Professional performance	

*N.s.* no significant relations identified

Our hypotheses on homophily of clinical attitudes and presence on opinion leaders were confirmed. This suggests that professional views in a practice team have impact on its performance. Although we are unaware of previous research which specifically investigated network homophily on positive attitudes of treatment outcomes of patients, this effect is in line with several other studies [28]. Homophily may be caused by selection of similar contacts or can be induced by repeated contacts with individuals with certain attributes [25]. Given that practices contain a heterogeneous group of health professionals, attitude-based homophily in our practices is unlikely to be caused by selection of similar contacts and may more likely have resulted by being part of a common context and by mutually experienced social influences. Positive effects of opinion leaders are in line with several studies. However, mixed findings on opinion leaders have been noted in current literature. For example, educational interventions which involved opinion leaders had moderate effectiveness [28], with different effects within primary and secondary care identified as well. Relatively few studies focused on mechanisms by which opinion leaders assert their effectiveness, of which understanding is therefore still limited. However, mechanisms described include generating consensus [43], increasing the observability and reducing potential risk of new clinical behaviors [44], and promoting efficient learning [27].

Dissimilar to previous studies, we found no effect of network density, frequency of contact, and centrality of CVRM coordinators. This may indicate that network structure may have limited impact in general practices. Density and frequency of contact are theorized to have its effects as many ties and contacts can create higher levels of information sharing and provide more momentums for collaboration [45]. Several reasons may explain why no effect of density and frequency of contacts were found in this study. First, sizes of networks of practices in this study were rather small. It may be that a low density and frequency of contacts in small networks are already sufficient to influence its members. For example, in a practice with five health professionals, knowledge may spread more readily than in a practice containing 15 health professionals, in which more contact moments may be required before information has been conceived by all network members. Second, it is possible that wider networks of healthcare professionals (contacts with health professionals from outside the practice organization and possibly also contacts from the past) were also, or more, relevant for their behaviors and views. For example, external contacts with possible influences on the practice nurses may likely consist of contacts with the CVRM care group (which represents the organization of general practitioners to provide CVRM in the Netherlands according to the chronic care model, which arranges funding, monitors performance, and provides feedback). Also, many information sources on CVRM are nowadays readily available on the Internet. Possibly then, health professionals needed to rely less on information exchange with colleagues to obtain needed information. Third, results may have been influenced by the timing of measurement of information exchange. In this study, we focused on information which was not new to health professionals as implementation of adherence to CVRM guidelines has been targeted by several interventions in the Netherlands in the past. In networks in which information has had spread effectively, it is possible that a low frequency of contact is already sufficient to influence its members, leaving additional contact moments without additional value. More contacts then could even be disadvantageous as persons may waste time and effort on maintaining contacts which are unable to provide them with new information [46, 47]. As such, density and frequency of contact may still be relevant network characteristics for improving delivery of care and patient risk factors, but of which effects may be more relevant and observable at earlier stages of implementation and spread of new knowledge.

The well spread of, and adequate availability of, CVRM knowledge may also account for the non-significant differences between characteristics of general and specific information exchange networks, which were contrary to our expectations. It is difficult to compare this result to previous literature as, to our best knowledge, no other studies investigated such networks. However, if all or the majority of health professionals were already equipped with adequate knowledge, there may have been no need to employ different information exchange patterns for discussing decisions on individual patients. On the other hand, it may be that specific information exchange was not recognized as such. CVRM guidelines also provide information on specific patient groups, which may have led health professionals to consider communication on treatment for specific patients as general information exchange.

In contrast to other research [21, 22, 48], but in line with a study on general practitioners’ prescribing behavior [20], we found no effect of centrality of CVRM coordinators (often practice nurses) on any of the outcomes. Fattore et al. [20] provided several reasons for the lack of effect of centrality which may be applicable in this study as well. They considered Granovetter [47] and Burt’s [46] notion of non-redundancy for information capability of networks; performance is influenced by networks which contain high informational dissimilarity. When relationships are equal in terms of access to resources (e.g., one individual within a network knows what other individuals know), a lack of access to new information may result. Having more contacts then, does not increase knowledge and thus does not influence other outcomes [46, 47]. However, specific details of this study may also explain why we were unable to identify effects of centrality. In our sample, we found that a substantial number of practices had more than one CVRM coordinator, with varying consistency rates when two or three coordinators were present. We choose to term the most consistently chosen person as coordinator and analyzed total degree of these persons. This approach might have obscured the effect of centrality as it may be that other coordinators were actually present and that their centrality scores mattered as well. Possible reasons for appointing more than one coordinator may include part time working employees, or the combination of coordination with other tasks. It may also be noted that in this study, at least one coordinator was present in almost all practices so that we were unable to investigate possible effects of absence of CVRM coordinators.

Strengths of the study include the substantial number of participating practices, the use of medical record data, and the use of both health professional performance outcomes and patient health outcomes. Limitations of the study include the following. First, the observational design of this design does not allow for causal inferences. Second, hypotheses were tested repeatedly, which can increase risk for type I error rate. We did not correct for this repeated testing given the explorative aim of this study and as corrections for repeated testing can come with disadvantages such as risk of enhanced type II rate. Third, practices were designated to have an opinion leader when at least 60% of healthcare workers in a practice choose a particular person as opinion leader. This cut-off of 60% may seem fairly low. However, it may be argued that 60% counts as a substantial number as network sizes were rather small. For example, in a practice with five workers, this would mean that three workers would consider a fourth person as their opinion leader, leaving a single person “unaffected” by the presence of the opinion leader. Fourth, the questionnaires for mapping the networks were not validated against a criterion measure. However, previous research using similar questionnaires showed that these provided feasible measurements with substantial variation [37–39]. Fifth, caution is warranted to generalize results of this study to other networks than those of primary care for CVRM.

All together, we found no effects of network structure characteristics (density, frequency of contact, coordinator’ centrality), while effects of network member’s views (homophily of clinical attitudes and presence of opinion leaders) were related to performance. These findings may indicate that for primary care for CVRM views, or its “culture”, in general practices may be more important than network structures. However, this does not mean that network structure can be ignored because many other studies provided evidence of influence on diffusion of information and collaboration between individuals. In addition, in this study, possible explanations for the non-significant influences of network structure included the timing of measurement of information exchange patterns. Future research is needed to enhance understanding of network structure, network culture, and its causal order, of which no inferences can be made in this observational study and on which disagreement exists in current literature.

Moreover, our results signal the potential of social networks to be taken into account in further attempts to improve the implementation of evidence-based care for CVRM. Future efforts may focus on individuals who are perceived as opinion leaders in practices and use these persons as conduits for disseminating new knowledge or as providers of assistance for adhering to guidelines for providing optimal care. The use of opinion leaders to promote evidence-based practice is not new. A Cochrane review [28] described that opinion leaders may be successful but that their identification, roles, and effectiveness showed a great variety, with different effects identified in primary and secondary care settings as well. One study noted, in line with our results, that opinion leaders could not be identified in every practice (47). Also, opinion leaders may not remain the same over a longer time period [49]. Therefore, further research focusing on the specific roles and influences of opinion leaders in primary care for CVRM is likely needed in order to identify and formulate optimal strategies for using opinion leaders to improve CVRM.

Our results on homophily indicate the importance of particular and common views towards treatment goals in CVRM, which is consistent with the notion that CVRM is a team effort. Implications may be two-sided. First, our results showed that a substantial number of health professionals did not value treatment target achievement and indicated a general agreement of this negative attitude within practices. The negative relation with SBP outcomes of patients may indicate that it is this negative attitude towards treatment targets which needs to be targeted in future interventions. Second, the effect of homophily underscores the importance of a common vision on CVRM within practices. Future interventions may therefore be more effective if they target the development, or strengthening, of a common (and possibly positive) vision on CVRM within practices. Possible examples of such interventions may include the use of opinion leaders, or incorporate approaches such as relational coordination, or reciprocal learning. The latter two represent mutual reinforcement interaction processes and learning as continuous and joint give-and-take process, respectively, and have been associated with improved quality of care and patient outcomes in settings which require efficient team work [50]. Future research is needed to assess the effectiveness of such interventions, or to identify other approaches which target shared conceptions and views within networks.

## Conclusions

This observational study aimed to find additional determinants for CVRM using social network analysis. Prevailing views in primary care teams, but no other social network characteristics, in information exchange networks of health professionals from practices were related to delivery of evidence-based health care.

## Abbreviations

CVD: Cardiovascular disease
CVRM: Cardiovascular risk management
LDL: Low-density lipoprotein
RCT: Randomized controlled trial
SBP: Systolic blood pressure
TICD: Tailored implementation for chronic diseases
